# The benefit of MR‐only radiotherapy treatment planning for anal and rectal cancers: A planning study

**DOI:** 10.1002/acm2.13423

**Published:** 2021-10-22

**Authors:** David Bird, Michael G. Nix, Hazel McCallum, Mark Teo, Alexandra Gilbert, Nathalie Casanova, Rachel Cooper, David L. Buckley, David Sebag‐Montefiore, Richard Speight, Bashar Al‐Qaisieh, Ann M. Henry

**Affiliations:** ^1^ Leeds Cancer Centre Leeds Teaching Hospitals NHS Trust Leeds UK; ^2^ Radiotherapy Research Group Leeds Institute of Medical Research Leeds UK; ^3^ Northern Centre for Cancer Care Newcastle upon Tyne Hospitals NHS Foundation Trust Newcastle upon Tyne UK; ^4^ Centre for Cancer Newcastle University Newcastle upon Tyne UK; ^5^ Biomedical Imaging University of Leeds Leeds United Kingdom

**Keywords:** MRI‐only planning, MR‐only

## Abstract

**Introduction:**

Limited evidence exists showing the benefit of magnetic resonance (MR)‐only radiotherapy treatment planning for anal and rectal cancers. This study aims to assess the impact of MR‐only planning on target volumes (TVs) and treatment plan doses to organs at risks (OARs) for anal and rectal cancers versus a computed tomography (CT)‐only pathway.

**Materials and methods:**

Forty‐six patients (29 rectum and 17 anus) undergoing preoperative or radical external beam radiotherapy received CT and T2 MR simulation. TV and OARs were delineated on CT and MR, and volumetric arc therapy treatment plans were optimized independently (53.2 Gy/28 fractions for anus, 45 Gy/25 fractions for rectum). Further treatment plans assessed gross tumor volume (GTV) dose escalation. Differences in TV volumes and OAR doses, in terms of Vx Gy (organ volume (%) receiving x dose (Gy)), were assessed.

**Results:**

MR GTV and primary planning TV (PTV) volumes systematically reduced by 13 cc and 98 cc (anus) and 44 cc and 109 cc (rectum) respectively compared to CT volumes. Statistically significant OAR dose reductions versus CT were found for bladder and uterus (rectum) and bladder, penile bulb, and genitalia (anus). With GTV boosting, statistically significant dose reductions were found for sigmoid, small bowel, vagina, and penile bulb (rectum) and vagina (anus).

**Conclusion:**

Our findings provide evidence that the introduction of MR (whether through MR‐only or CT‐MR pathways) to radiotherapy treatment planning for anal and rectal cancers has the potential to improve treatments. MR‐related OAR dose reductions may translate into less treatment‐related toxicity for patients or greater ability to dose escalate.

## 1 INTRODUCTION

Magnetic resonance imaging (MRI)‐only radiotherapy treatment planning is the use of an MRI scan alone to plan radiotherapy treatments. These techniques require the generation of a “synthetic‐computed tomography (CT)” (computer generated) dataset as MRI does not directly provide the patient density information required to allow dose calculation that is usually obtained from CT.^1^, ^2^, ^3^ MR‐only planning techniques have developed considerably in recent years, with commercial synthetic‐CT (sCT) solutions now available and specialist centers treating prostate cancers.^1^, ^2^, ^3^ However, a remaining challenge to wide‐spread adoption is the lack of evidence within the literature demonstrating the impact of MR‐only radiotherapy treatment planning to patients, in terms of improving treatments compared to standard pathways.^1^

To the authors' knowledge, only one study has assessed the impact of MR‐only radiotherapy treatment planning on patient outcomes; finding prostate treatment acute outcomes were similar to a CT‐MR pathway.^4^ For anal and rectal cancers, there is no evidence in the literature quantifying the impact of MR‐only radiotherapy treatment planning to patient treatments.

It is difficult to assess the impact of MR‐only radiotherapy treatment planning as standard pathways in routine clinical use include CT‐only or CT‐MR pretreatment imaging. CT‐only radiotherapy treatment planning pathways are common in many centers, for example in the UK, where dedicated radiotherapy MR provision is relatively scarce.^5^ However, where the MR simulation resources are available, CT‐MR pathways are the preferred option.^6^ The central hypothesis for using MRI in the radiotherapy treatment planning process, whether in a CT‐MR or MR‐only pathway, is that the improved soft‐tissue contrast of MR allows improved visualization of target volumes (TVs).^7^, ^8^ For anal and rectal cancers, it is hypothesized this could lead to more accurate definition of tumors and therefore reduced radiotherapy TVs. This is supported by diagnostic anal and rectal study findings of net reductions in tumor volume when delineated on MR versus CT.^9^, ^10^

Comparing MR‐only to CT‐MR pathways, there are a number of benefits which include reduced CT scanning, streamlining clinical workflows, and removing CT‐MR registration uncertainties.^7^, ^8^, ^11^ As well as the logistical and practical advantages, it is the removal of systematic CT‐MR registration errors^12^ which could further improve patient treatments.

Here, we aim to quantify the impact of MR‐only radiotherapy treatment planning on TVs and treatment plan doses to organ at risk (OAR) for anal and rectal cancer treatments when compared to a routine CT‐only simulation pathway. We hypothesize that reduced MR‐only TVs should result in treatment plans with reduced OAR doses if TV coverage is maintained. By comparing MR‐only and CT‐only pathways, we are assessing both the benefit of including MR in the treatment pathway, which is also true of a CT‐MR pathway and the benefit of removing CT‐MR pathway registration uncertainties. We also hypothesize that gross tumor volume (GTV) dose escalation planning^13^, ^14^, ^15^ would enhance the benefit from MR‐only versus CT‐only planning due to the reduced volume of GTVs delineated on MR versus CT.

## 2 METHODS

### 2.1 Data collection

This study recruited 46 patients with anal and rectal cancer from a single center; 29 rectum and 17 anus; 24 male and 22 female, who were due to undergo preoperative or radical volumetric arc therapy (VMAT) external beam radiotherapy. Exclusion criteria included patients with contra‐indications to MR. Patient demographics and staging can be seen in Appendix A, Table A1. This study is part of a wider MR‐only radiotherapy study: “Mri‐only treAtmeNT planning for Anal and Rectal cAncer radiotherapy” (MANTA‐RAY), research ethics committee reference: 18/LO/1298, ISRCTN Registry: ISRCTN82734641.

All patients received CT and T2‐SPACE MR simulation in the radiotherapy treatment position with matched bladder filling and immobilization protocols. CT and MRI scan parameters can be seen in Appendix A, Table A2. For MR simulation, coil bridges were used to prevent the coils from deforming the patient skin position. As MR simulation was for research purposes it was scheduled for a time when the patient had a clinical appointment, and the MR scanner was available. The mean time between planning CT and MR data acquisition was 15.1 days. The timings of the MR scans can be seen in Appendix A, Table A1, where in total 41% of MR scans were acquired prior to treatment starting, 69% were acquired by the end of week 1 of treatment (fraction 5), and 95% were acquired by the end of week 2 of treatment (fraction 10). MR scans were rigidly registered by an experienced clinical scientist specializing in image registration, focussing on the rectum and anal canal, to their paired CT datasets using the mutual information registration algorithm in Raystation 8b (RaySearch Laboratories, Stockholm, Sweden). MRs were resampled to the CT frame of reference using Raystation 8b's standard tri‐linear resampling. A synthetic‐CT (sCT) scan was generated from each patient's T2‐SPACE MR scan using a deep learning‐based cGAN sCT model. The cGAN sCT model and the CT and MR acquisition parameters have been previously described in the literature.^16^

### 2.2 TV and OAR delineation

All TVs (defined in Table 1) were delineated on CT and T2 MR simulation scans separately according to our center's clinical protocol, apart from GTVBoost volumes which were chosen according to clinical trials assessing GTV dose escalation.^13^, ^14^, ^15^ All patients had diagnostic MR available, and patients with anal cancer additionally had diagnostic PET‐CT available to assist delineations through side‐by‐side comparisons to the planning scan as per our center's clinical protocol. These diagnostic scans were unsuitable for registration and further use in the study.

**TABLE 1:** Target volume delineations and their definitions for anal and rectal cancers, including gross tumor volume (GTV), nodal gross tumor volume (GTVN), boost gross tumor volume (GTVBoost), primary clinical target volume (CTVA), elective clinical target volume (CTVB/E), nodal clinical target volume (CTVN), final clinical target volume (CTVF), primary planning target volume (PTV/PTVA), nodal planning target volume (PTVN) and elective planning target volume (PTVE)

Anus		Rectum	
Target volume	Definition	Target volume	Definition
GTV	Macroscopic primary tumor only	GTV	CT: Macroscopic primary tumor extended to whole lumen and identified nodal tumors MR: Macroscopic primary tumor only
CTVA	T1/2: GTV + 1.0 cm enlarged to include the whole of the anal canal and external sphincters	CTVA	GTV + 1.0 cm
	T3/4: GTV + 1.5 cm enlarged to include the whole of the anal canal and external sphincters	CTVB	Elective anatomically defined volume including the mesorectum, pre‐sacral, internal iliac and pelvic side‐wall nodes
PTVA	CTVA + 1.0 cm	CTVF	CTVA + CTVB
GTVBoost	GTV + 0.5 cm	PTV	CTVF + 1.5 cm (anterior) and 1 cm (all other directions)
GTVN	Identified nodal tumors	GTVBoost	GTV + 1.0 cm
CTVN	GTVN + 0.5 cm	X	
PTVN	CTVN + 0.5 cm		
CTVE	Elective anatomically defined volume including the mesorectal, bilateral inguinal, internal, and external iliac nodes to 2 cm above the lower border of sacroiliac joints, presacral nodes (or 1.5 cm superior from the most superior GTV)		
PTVE	CTVE + 0.5 cm		

CT TVs, as used for each patient's clinical treatment, were delineated by the treating consultant clinical oncologist. MR GTV delineations were undertaken by one of three consultant clinical oncologists specializing in anal and rectal cancer treatments with experience of interpreting T2 MR sequences for ano‐rectal tumor delineations. MR GTVs were undertaken 2+ months after CT delineations to avoid potential recollection bias. All GTV delineations were undertaken using all clinically available information, apart from the other scan (CT or MRI). An experienced clinical scientist specializing in radiotherapy imaging created all other MR TVs through expansions and manual adjustments, all matching the CT clinical pathway (Table 1). For elective CTVs (rectum and anus), the CT CTV was rigidly transferred to the MR and manually adjusted to anatomical boundaries, accounting for anatomical changes between CT and MR, for example differences in the mesorectum boundary.

OARs were delineated on CT and MR by experienced dosimetrists and included bladder, small bowel, sigmoid, penile bulb, vagina, uterus (rectum and anus), bowel cavity (rectum only), femoral heads, and genitalia (anus only). All OARs were assessed and amended as required by an experienced clinical scientist to ensure accuracy. Genitalia OARs (delineated from anatomical landmarks) were delineated on CT and rigidly transferred to MR to ensure consistency. Penile bulb, vagina, and uterus were delineated on MR and transferred to CT (rigid registration: penile bulb, deformable registration: vagina and uterus). These registrations were undertaken and validated through visual assessment by an experienced physicist who specializes in MR‐CT registrations. The decision to transfer the OARs from MR was made because of the poor visualization of these OARs on CT. All MR TVs and OARs were rigidly transferred to the sCT from the MR to allow radiotherapy treatment planning to occur.

### 2.3 TV analysis

Volumetric and positional TV analyses were undertaken. The positional analysis compared the overlap of CT and MR volumes using sensitivity and specificity measures—the volume overlap between CT and MR contours, as a percentage of the volume of CT (sensitivity) or MR (specificity) respectively.

### 2.4 Radiotherapy treatment planning

VMAT plans were created and optimized for each patient's CT and sCT scan independently by a single experienced clinical scientist following the center's clinical protocol. Plans were optimized for the delineated TVs and OARs, in Raystation 8b, using the collapsed cone photon algorithm on a dose grid of 3 × 3 × 3 mm^3^ and beam arrangements seen in Appendix A, Table A3. Rectum plans were prescribed as 45 Gy in 25 fractions to the primary PTV, and anus plans were prescribed a three dose level technique with 53.2 Gy, 50.4 Gy, and 40.0 Gy in 28 fractions to the primary, nodal, and elective PTVs, respectively (subsequently referred to as “standard” plans). To increase the homogeneity of prescription doses, we opted to standardize the dose prescription for each cancer site (in practice some patients received 25 Gy in 5 for rectal cancers and 50.4 Gy for T1/2N0 anal cancers).

Dose escalation plans (“Boost” plans) were also generated to assess the impact of dose escalation to GTV‐based (“GTVBoost”) structures with 61.6 Gy and 55 Gy prescribed for anus and rectum, respectively. GTVBoost prescription doses were chosen according to clinical trials assessing GTV dose escalation.^13^, ^14^, ^15^ Boost plans were created by copying each standard plan, adding optimization constraints and objectives for the GTVBoost contour and re‐optimizing the plan. All treatment plan optimization parameters and clinical objectives can be seen in Appendix A, Tables A4 and A5.

All plans were optimized to meet target coverage constraints while minimizing OAR doses. The planning protocol was adapted to include a high mandatory coverage goal for all PTVs, with the rationale that high target coverage prevents subjective, plan specific, local areas of poor PTV coverage within the plan objectives which may impact OAR dose reductions.

### 2.5 VMAT plan analysis

As all plans had strict TV coverage criteria, plan assessment focussed on the dosimetric differences to OARs when TV coverage was achieved. Each OAR was assessed in terms of the Vx (%), the volume of the organ as a percentage of the total organ volume, receiving x Gy in dose. DVH statistics were collected for 95%, 90%, 80%, 70%, and 60% of the prescription dose for each standard plan and compared between CT and MR. These dose levels were chosen to allow a more comprehensive analysis of the dose‐volume relationship for each OAR. Dose levels lower than 60% of the prescription dose were not assessed. For boost plans, DVH assessments focussed on the dose levels introduced by the GTVboost prescription (52.25 Gy, 49.5 Gy, and 45 Gy [rectum] and 58.5 Gy, 55.4 Gy, and 53.2 Gy [anus]).

For standard and boost plans, collected DVH statistics were filtered such that if both the CT and MR DVH statistic (Vx) were ≤1% then the statistic was removed from the analysis. This removed cases where the TV and OAR were separated sufficiently that the OAR was not receiving that dose level on either plan. In all cases, the femoral heads received a dose less than 60% of the prescription and consequently were removed from the analysis.

### 2.6 Statistical analysis

Linear mixed effects (LME) models in STATA^17^ were applied to TV volume and OAR dose analyses to establish statistically significant differences in TVs and OAR doses attributable to the change in modality, CT, and MR. Separate models were applied to each cancer site cohort (anus and rectum) and TV/OAR dose level individually. DVH statistic differences were only modeled if five or more patient's results were present. TV LME models used volume (cc) as the dependant variable, modality (CT and MR), sex (male and female), and staging (1, 2, 3, or 4) as fixed effects independent variables. The OAR dose LME models used DVH statistic, Vx, as the dependant variable, modality (CT and MR), sex (male and female), and organ volume as fixed effect independent variables. Patient was a random effect independent variable in all models. Organ volume was included to account for impact of variations in organ volume (for example the bladder) on the DVH statistics.

## 3 RESULTS

**FIGURE 1:**
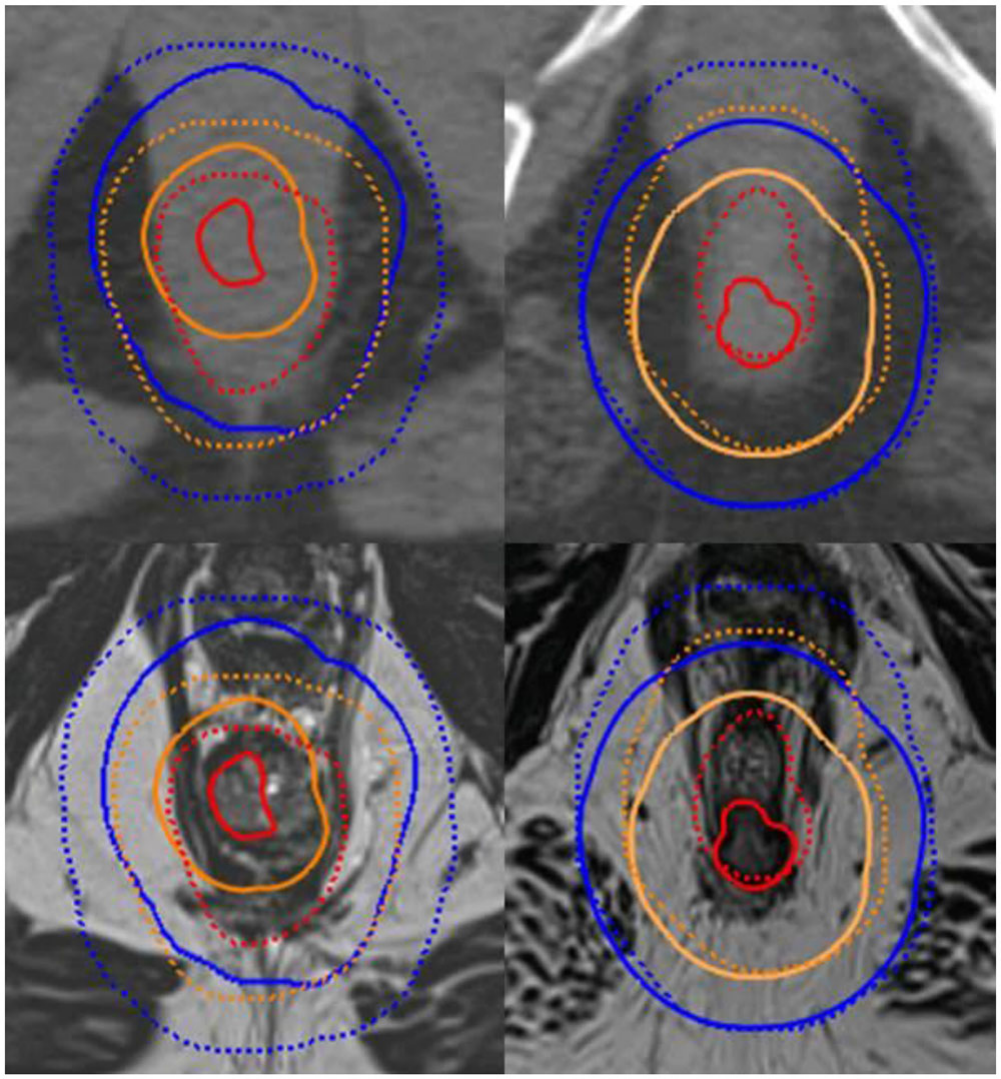
Comparison of anus (right) and rectum (left) cancer GTVs (red), CTVAs (orange), and primary PTVs (blue) for MR (bold) versus CT (dotted) delineations on CT (top) and T2 SPACE MR (bottom) datasets

**FIGURE 2:**
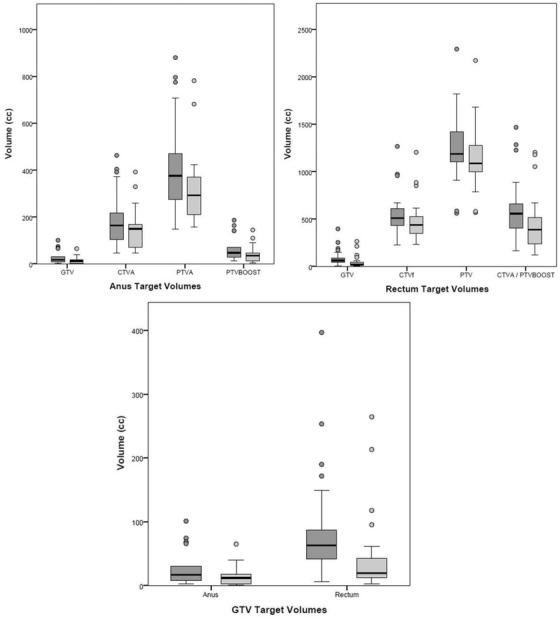
Box‐plots comparing the volumes of treatment target volumes for anal cancers (top left), rectal cancers (top right), and all GTVs (bottom middle) on CT (dark grey) and MR (light grey) including the median, interquartile range, and outlier values.

TV comparisons for both anal and rectal cancers found a statistically significant systematic reduction in MR GTV (12.6 cc and 43.6 cc respectively), primary PTV (98.1 cc and 109.1 cc respectively), and GTVBoost (22.3 cc and 95.2 cc respectively) volumes compared to CT. Table 2 shows the MR TV volume differences versus CT, their statistical significances and the positional overlap between MR and CT. Figure 1 shows a visual example of the changes in GTV, CTVA, and primary PTV between MR and CT for a single anus and rectum cancer case. Figure 2 shows box plots of the volumes of the anal and rectal radiotherapy TVs on CT an MR.

**TABLE 2:** The magnetic resonance (MR) target volume (TV) differences in volume compared to computed tomography (CT) and the mean sensitivity and specificity overlap for each target volume between MR and CT over the whole patient cohort, where effect size is the systematic difference between MR and CT volumes (a negative value indicates that MR is smaller than CT). Bold effect size values indicate statistically significant confidence intervals

				Mean overlap	
		MR volume effect size versus CT (95% confidence intervals) (cc)	MR volume effect size versus CT (%)	Sensitivity (% of CT)	Specificity (% of MR)
**Rectum**	**GTV**	**−43.6 (−54.8 to** −**32.5)**	**−56.5**	36.4	93.7
	**CTVA**	**−95.2 (−116.3 to −74.3)**	**−37.1**	57.8	95.2
	**CTVB**	**−11.7 (−17.1 to −6.3)**	**−2.6**	94.5	97.6
	**CTVF**	**−66.0 (−80.7 to −51.4)**	**−12.4**	85.7	98.3
	**PTVA**	**−109.1 (−131.1 to −87.2)**	**−8.6**	90.3	99.0
	**GTVBoost**	**−95.2 (−116.3 to −74.3)**	**−28.2**	57.8	95.2
**Anus**	**GTV**	**−12.6 (−19.5 to −5.7)**	**−46.6**	32.5	80.3
	**CTVA**	**−47.7 (−72.4 to −22.9)**	**−20.7**	66.0	87.1
	**PTVA**	**−98.1 (−146.1 to −50.1)**	**−19.3**	72.4	92.0
	**GTVN**	−3.0 (−7.6 to 1.5)	−15.7	53.5	73.0
	**CTVN**	−9.2 (−20.3 to 1.9)	−12.5	70.7	84.5
	**PTVN**	**−24.5 (−47.0 to −2.1)**	**−16.5**	74.4	91.9
	**CTVE‐all**	**−35.0 (−56.6 to −13.4)**	**−4.0**	89.8	94.7
	**PTVE‐all**	**−106.8 (−144.7 to −68.9)**	**−5.9**	88.1	94.2
	**GTVBoost**	**−22.3 (−33.7 to −10.9)**	**−34.4**	46.9	81.8

Abbreviations: CTVA, primary clinical target volume; CTVB/E, elective clinical target volume; CTVF, final clinical target volume; CTVN, nodal clinical target volume; GTV, gross tumor volume; GTVBoost, boost gross tumor volume; GTVN, nodal gross tumor volume; PTV/PTVA, primary planning target volume; PTVE, elective planning target volume; PTVN, nodal planning target volume.

For anus plans, statistically significant dosimetric reductions were found on MR (vs. CT) plans for the bladder (3.8%), penile bulb (∼10%), and genitalia (∼4%). Systematic dose reductions that had not reached statistical significance were also found for the vagina (∼13%). For rectum plans, statistically significant dosimetric reductions were found on MR (vs. CT) plans for the bladder (∼5%) and uterus (∼13%). Systematic dose reductions that had not reached statistical significance were found for the penile bulb (∼6%). Table 3 shows the dosimetric differences to OARs in standard plans between MR and CT.

**TABLE 3:** The magnetic resonance (MR) dosimetric differences to organs at risks (OARs) in standard plans for anal and rectal cancer treatments, where volume effect size is the systematic difference in volume of each organ receiving x Gy of dose on MR versus computed tomography (CT) (a negative value indicated a lower dose on MR compared to CT). Bold effect size values indicate statistically significant confidence intervals. “Number of patients” is the number of patients whose DVH statistics were >1% on both CT and MR and therefore included in the analysis

		Anus		Rectum	
Standard Plans	Dose level	Number of patients	Vx effect size (95% confidence intervals) (%)	Number of patients	Vx effect size (95% confidence intervals) (%)
**Bladder**	**V95%**				
	**V90%**				
	**V80%**	7	−0.6 (−5.6 to 4.4)	28	−**5.3 (**−**8.2 to** −**2.4)**
	**V70%**	17	−**3.8 (**−**6.4 to** −**1.2)**	28	−**5.2 (**−**8.2 to** −**2.3)**
	**V60%**	17	−4.1 (−8.6 to 0.3)	28	−**5.2 (**−**8.2 to** −**2.2)**
**Small bowel**	**V95%**	0	–	23	−1.0 (−5.4 to 3.4)
	**V90%**	1	–	23	−0.9 (−5.6 to 3.7)
	**V80%**	3	–	23	−0.9 (−5.6 to 3.7)
	**V70%**	13	2.0 (−2.0 to 5.9)	23	−0.8 (−6.1 to 4.4)
	**V60%**	14	5.0 (−0.7 to 10.7)	23	−0.8 (−6.7 to 5.0)
**Sigmoid**	**V95%**	2	–	29	−3.0 (−7.1 to 1.1)
	**V90%**	3	–	29	−2.7 (−6.9 to 1.5)
	**V80%**	5	−1.5 (−6.5 to 3.5)	29	−1.8 (−6.3 to 2.7)
	**V70%**	17	−2.7 (−9.6 to 4.3)	29	−1.0 (−5.7 to 3.7)
	**V60%**	17	−2.5 (−11.1 to 6.0)	29	−0.6 (−5.7 to 4.5)
**Vagina**	**V95%**	8	−12.5 (−29.2 to 4.2)	14	0.7 (−1.3 to 2.6)
	**V90%**	8	−12.9 (−28.9 to 3.2)	14	0.5 (−1.3 to 2.3)
	**V80%**	8	−16.0 (−33.4 to 1.4)	14	0.1 (−1.3 to 1.6)
	**V70%**	8	−3.5 (−7.7 to 0.7)	14	0.2 (−1.1 to 1.5)
	**V60%**	8	1.1 (−0.5 to 2.7)	14	0.7 (−0.7 to 2.1)
**Uterus**	**V95%**	2	–	13	−**15.9 (**−**24.4 to** −**7.4)**
	**V90%**	2	–	13	−**14.9 (**−**23.8 to** −**6.1)**
	**V80%**	3	–	13	−**13.8 (**−**22.6 to** −**4.9)**
	**V70%**	7	−10.4 (−27.3 to 6.6)	13	−**12.8 (**−**21.3 to** −**4.3)**
	**V60%**	7	2.6 (−24.3 to 29.6)	13	−**11.8 (**−**20.3 to** −**3.2)**
**Penile Bulb**	**V95%**	9	−**11.2 (19.9 to** −**2.5)**	6	−7.3 (−27.2 to 12.5)
	**V90%**	9	−**9.6 (**−**16.4 to** −**2.7)**	9	−5.4 (−18.4 to 7.6)
	**V80%**	9	−**8.3 (**−**15.5 to** −**1.2)**	9	−6.0 (−18.2 to 6.2)
	**V70%**	9	−**8.4 (**−**16.2 to** −**0.7)**	9	−7.3 (−18.8 to 4.2)
	**V60%**	9	−**8.8 (**−**16.8 to** −**0.7)**	10	−9.7 (−21.5 to 2.1)
**Genitalia**	**V95%**	5	−4.8 (−11.0 to 1.3)	–	
	**V90%**	5	−5.2 (−12.0 to 1.6)		
	**V80%**	6	−5.1 (−11.5 to 1.3)		
	**V70%**	11	−**4.0 (**−**7.5 to** −**0.5)**		
	**V60%**	17	−**3.5 (**−**5.7 to** −**1.3)**		

For rectum boost plans, statistically significant dosimetric reductions were found for the bladder (4.6%), small bowel (∼3.5%), sigmoid (∼6%), vagina (13.6%), uterus (∼20%), and penile bulb (11.5%). For anus plans, statistically significant dosimetric reductions were found for the vagina (4.4%) and penile bulb (15.4%). Table 4 shows the dosimetric differences to organs in boost plans between CT and MR.

**TABLE 4:** The MR dosimetric differences to organs at risks (OARs) in boost plans for anal and rectal cancer treatments, where volume effect size is the systematic difference in volume of each organ receiving x Gy of dose on magnetic resonance (MR) versus computed tomography (CT) (a negative value indicated a lower dose on MR compared to CT). Bold effect size values indicate statistically significant confidence intervals. “Number of patients” is the number of patients whose dose‐volume histogram (DVH) statistics were <1% on both CT and MR and therefore included in the analysis

	Anus			Rectum		
Boost Plans	Dose level	Number of patients	Vx Effect size (95% confidence intervals) (%)	Dose level	Number of patients	Vx effect size (95% confidence intervals) (%)
Bladder						
	V55.4	0	–	V49.5	11	−3.0 (−6.1 to 0.1)
	V53.2	2	–	V45	27	−**4.6 (**−**7.6 to** −**1.7)**
Small bowel	V58.5	0	–	V52.25	8	−**3.4 (5.9 to** −**0.8)**
	V55.4	0	–	V49.5	8	−**3.6 (**−**6.7 to** −**0.5)**
	V53.2	0	–	V45	23	−1.0 (−3.9 to 1.9)
Sigmoid	V58.5	0	–	V52.25	16	−**6.4 (**−**9.1 to** −**3.7)**
	V55.4	0	–	V49.5	16	−**6.4 (**−**9.6 to** −**3.3)**
	V53.2	1	–	V45	29	−**4.6 (8.9 to** −**0.4)**
Vagina	V58.5	7	−**4.4 (**−**8.0 to** −**0.8)**	V52.25	13	−**13.6 (**−**26.3 to** −**0.9)**
	V55.4	7	−8.7 (−22.0 to 4.7)	V49.5	14	−6.9 (−17.1 to 3.2)
	V53.2	8	−11.1 (−28.3 to 6.0)	V45	14	−1.7 (−5.7 to 2.2)
Uterus	V58.5	0	–	V52.25	11	−**19.8 (**−**30.7 to** −**9.0)**
	V55.4	1	–	V49.5	12	−**21.9 (**−**32.7 to** −**11.2)**
	V53.2	2	–	V45	13	−**19.6 (**−**26.9 to** − **12.3)**
Penile Bulb	V58.5	3	–	V52.25	3	–
	V55.4	7	−22.4 (−49.2 to 4.4)	V49.5	4	–
	V53.2	8	−**15.4 (**−**30.3 to** −**0.5)**	V45	6	−**11.5 (**−**34.3 to** −**11.3)**
Genitalia	V58.5	2	–	–		
	V55.4	3	–			
	V53.2	4	–			

## 4 DISCUSSION

A challenge to wide‐spread adoption of MR‐only radiotherapy treatment planning is the lack of evidence within the literature demonstrating its benefit in terms of improving treatments. Here, we provide evidence that utilizing an MR‐only radiotherapy pathway for anal and rectal cancers makes statistically significant changes to TV volumes and treatment plan OAR doses, in terms of reductions in volume (∼100 cc for PTV/PTVA) and dose‐volume (5%–20%) compared to a CT‐only pathway. These TV and treatment plan changes can be considered evidence of benefit as smaller TVs result in less irradiated tissue, and lower normal tissue doses can be expected to lead to reduced organ toxicities.^18^ It is important to recognize that while we have compared an MR‐only radiotherapy pathway to a CT‐only pathway, many centers with radiotherapy MR provision employ a CT‐MR pathway. Our findings are more difficult to apply to CT‐MR pathways which, compared to MR‐only, introduce co‐registration errors. As such the treatment changes, we present here would likely be smaller for a CT‐MR pathway. However, our findings can also broadly be applied to MR‐CT pathways and therefore add to a growing body of evidence that the introduction of MR can improve treatments, whether through MR‐only or CT‐MR pathways, which has not previously been shown in the literature in these terms.

Our findings showed that the improved soft tissue visualization of MR translated into reduced TV volumes for both anal and rectal cancers. GTV volumes were reduced significantly as suggested by diagnostic CT versus MR comparisons in the literature,^9^, ^10^ and this translated through to reductions in primary PTV volumes. The resultant ∼100 cc reduction in PTV volume is a significant amount of tissue which will be spared a high (prescription level) dose. Both cancer sites also saw significant reductions in anatomically defined CTVB/E volumes from CT to MR. A visual assessment of the CTVs showed that this was due to an improvement in tissue visualization at the mesorectum anterior border, where it is difficult to define the mesorectum, vagina, and seminal vesicles borders on CT. This soft‐tissue contrast improvement has the potential to also improve clinician confidence, speed, and inter‐observer variability when delineating, although assessing this was beyond the scope of this study. The GTVBoost volume reductions also add evidence that MR‐based radiotherapy treatment planning may have a greater impact on boost plans. Our positional differences between MR and CT delineated TVs showed the specificity of TVs (overlap as a percentage of the MR volume) was much higher than the sensitivity (overlap as a percentage of the CT volume), suggesting that MR volumes are not only smaller than CT, but also predominantly within the CT volumes which adds strength to the hypothesis that MR improves the visualization of TVs. It is notable that our delineation protocol for rectum GTVs varied between CT and MR, where CT GTVs included the whole lumen but MR GTVs included the visible tumor only. This was due to the soft tissue contrast of MR enabling a systematic change in delineation protocol through improved visualization, and it is the impact of this improved visualization that we have quantified here.

Our standard treatment plan analysis found statistically significant reductions in OAR doses for both cancer sites. This provides evidence that MR‐only radiotherapy treatment planning makes a quantitatively significant improvement to treatment plans compared to CT‐only pathways. The OAR dose changes that we saw here are logical. For standard plans we saw that the organs closest to the primary GTV—the sexual organs for anal cancers,^19^ and the bladder and uterus for rectum cancers had statistically significant systematic dose reductions, whereas we saw no change to the small bowel dose which is predominantly the organ furthest from the GTV. There were also a number of organs that had systematic dose reductions that had not reached statistical significance. While less definitive, these findings should also be viewed positively and suggest that there may be additional benefit in a larger population. Our dose escalation (boost) plan analysis also suggests that MR boost plans were able to improve the sparing of OARs very close to the primary GTV, for example the vagina and penile bulb for anal cancers, but that for rectal cancers there was a much wider dose reduction to the majority of OARs, including small bowel and sigmoid. This can be explained by the much larger GTVBoost volume for rectal cancers compared to anal cancers, the central position of the rectum in the pelvic cavity, and the differences in the elective CTV standard plan dose prescription between rectal and anal cancers.

There are limitations to this study. It is known that some tumor shrinkage occurs during treatment as shown by Van den Begin^20^ who found for rectal cancers MRI GTVs versus a pretreatment baseline volume that tumor shrinkage of up to 10% after 1 week (five fractions) and 26% after 2 weeks (10 fractions) can occur. Logistical challenges meant it was necessary to accept acquiring MR scans after patient treatments had started, and this had the potential to bias our findings. However, to assess this, we stratified our CT to MR GTV volume findings by MR acquisition date and found that for both anal and rectal cancers, there was no correlation between MR scan timing and average GTV reduction (pretreatment = −53%, week 1 = −44%, and week 2 = −56%). It is possible that some component of the tumor reduction we identified here has been caused by treatment; however, the GTV reductions seen here are much larger than those demonstrated due to treatment by Van den Begin, and consequently, it is more likely that the large reduction in GTV caused by improved MR tissue visualization substantially outweighs the impact of treatment GTV changes.

It is possible that the OAR dose reductions found here may be insufficient to produce meaningful toxicity reductions. Future work would benefit from assessing the impact of these dose reductions on normal tissue complication probability for the organs highlighted here. However, this is a non‐trivial assessment to undertake, and as such it was outside the scope of this study to assess dose‐toxicity relationships. We also did not assess OAR changes in relation to the position of the tumor; however our use of filtering DVH statistic was designed to reduce the impact of this on our analysis. Further work would be beneficial to investigate whether it is possible to prospectively identify patients who would benefit most from MR‐only planning, prior to simulation. A challenge with planning studies is ensuring that treatment plan differences are not due to inter‐operator variability in delineations and planning. Here, there was the potential for inter‐operator variability as the clinicians delineating GTVs differed between CT and MR. However, we aimed to avoid significant inter‐operator variability through delineations being undertaken by experienced consultants, following our local clinical protocols with additional training, stricter planning constraints, and oversight by a single physicist for consistency. The simulation protocols also minimized our OAR volume differences and the uncertainty this can cause between CT and MR as seen in Appendix A, Table A6.

There is also an argument that reducing TVs due to a change in imaging modality could have a negative effect on tumor control probability as our understanding of required treatment dose levels stems from CT‐based targets and the reduction of the TVs is in essence removing implicit margins caused by a lack of contrast on CT. This argument highlights the need for caution when assessing new techniques such as MR‐only planning.

Our findings suggest that MR‐only radiotherapy treatment planning can be considered to be an improvement in the personalization of radiotherapy treatments, compared to CT‐only, as it allows clearer visualization of individual patient anatomy. Here, we aimed to assess the impact of MR‐only radiotherapy treatment planning on TVs and treatment plan doses to OARs for anal and rectal cancers when compared to a routine CT‐only pathway. Our findings provide evidence that MR‐only radiotherapy treatment planning for anal and rectal cancers results in statistically significant reductions in TV volumes and reduced doses to a number of OARs. This suggests that patients could benefit from MR‐only (or CT‐MR) radiotherapy treatment planning with the potential for improved patient outcomes if OAR dose reductions translate into less treatment‐related toxicity or support GTV dose escalation.

## CONFLICT OF INTEREST

The authors declare that there is no conflict of interest that could be perceived as prejudicing the impartiality of the research reported.

## APPENDIX A

**TABLE A1:** Anal and rectal cancer patient demographics

		Anus (total 17)	Rectum (total 29)
Age	**Mean (range)**	**63 (47–76)**	**63 (38–78)**
Sex	Male	9	15
	Female	8	14
Staging	T1	2	1
	T2	5	11
	T3	9	16
	T4	1	1
MR scan acquired	Pretreatment	6	13
	Fraction 1–5	4	9
	Fraction 6–10	6	6
	Fraction 11–13	1	1

**TABLE A2:** computed tomography (CT) and magnetic resonance (MR) simulation scan parameters

MR	Make & Model	Siemens Aera 1.5 T
	Sequence	3D
	Resolution	0.9 × 0.9 × 1.5 mm
	Refocusing flip angle (°)	160
	TR (ms)	1600
	TE (ms)	211
	Bandwidth (Hz/px)	545
	Echo train length	134
	Field of view (superior‐inferior)	Inferior: 2 cm inferior of genitalia Superior: superior aspect of L5 vertebra or greater as required
	Field of view (Axial)	450 × 450 mm^2^
CT	Make & Model	Philips Brilliance Big Bore
	Resolution	1.2 × 1.2 × 2 mm
	kVp (kV)	120
	X‐ray tube current (mAs)	135

**TABLE A3:** Treatment plan beam arrangement and prescription parameters for standard plans

		Anus	Rectum
Modality		Photons	
Technique		VMAT	
Prescription type		“Average Dose”	
Isocenter Position		Center of PTV	
Energy (MV)		6MV FFF	
Beam arrangement		360° arc (starting at 180°)	
Beam optimization settings		Dual arc	
Standard Plan Prescription	PTV	53.2 Gy in 28 fractions	45 Gy in 25 fractions
	PTVN	50.4 Gy in 28 fractions	X
	PTVE	40 Gy in 28 fractions	X
Boost plan additional prescription	GTVBoost	61.6 Gy in 28 fractions	55 Gy in 25 fractions

Abbreviations: GTVBoost, boost gross tumor volume; PTV, primary planning target volume; PTVE, elective planning target volume; PTVN, nodal planning target volume; VMAT, volumetric arc therapy.

**TABLE A4:** Treatment plan initial optimization parameters for standard and boost plans for anal and rectal cancers

	Optimization objectives					
	Rectum			Anus		
	Target volume/OAR	Objective	Description	Target volume/OAR	Objective	Description
Standard plan	PTV	Min dose	45 Gy	PTV	Min dose	53.2 Gy
		Uniform dose	45 Gy		Uniform dose	53.2 Gy
	Patient external	Dose fall off	High: 45 Gy, Low: 25 Gy, Distance: 1.5 cm	PTVN ‐ PTV	Min dose	50.4 Gy
		Dose fall off	High: 25 Gy, Low: 0 Gy, Distance: 6.0 cm		Uniform Dose	50.4 Gy
	Bladder‐PTV	Max DVH	20.0 Gy to 30%		Dose fall off	High: 53.2 Gy, Low: 50.4 Gy, Distance: 2.0 cm
	Bowel cavity ‐PTV	Max DVH	20.0 Gy to 30%	PTVE ‐ (PTV+PTVN)	Min dose	40 Gy
	Penile bulb ‐ PTV	Max DVH	20 Gy to 35%		Dose fall off	High: 53.2 Gy, Low: 40.0 Gy, Distance: 2.0 cm
	Uterus ‐ PTV	Max DVH	20 Gy to 35%	Patient external	Dose fall off	High: 53.2 Gy, Low: 25 Gy, Distance: 1.5 cm
	Vagina ‐ PTV	Max DVH	20 Gy to 35%		Dose fall off	High: 53.2 Gy, Low: 0 Gy, Distance: 6.0 cm
	X			Bladder ‐ PTVs	Max DVH	20 Gy to 35%
				Small bowel ‐ PTVs	Max DVH	20 Gy to 35%
				Femoral heads ‐ PTVs	Max DVH	20 Gy to 35%
				Genitalia ‐ PTVs	Max DVH	20 Gy to 35%
				Penile bulb ‐ PTVs	Max DVH	20 Gy to 35%
				Uterus ‐ PTVs	Max DVH	20 Gy to 35%
				Vagina ‐ PTVs	Max DVH	20 Gy to 35%
Boost plan objectives	GTVBoost	Min dose	55.0 Gy	GTVBoost	Min dose	61.6 Gy
		Uniform dose	55.0 Gy		Uniform dose	61.6 Gy
	PTV	Dose fall off	High: 55.0 Gy, Low: 45.0 Gy, Distance: 1.0 cm	PTV	Dose fall off	High: 61.6 Gy, Low: 53.2 Gy, Distance: 1.0 cm

Abbreviations: GTVBoost, boost gross tumor volume; PTV, primary planning target volume; PTVE, elective planning target volume; PTVN, nodal planning target volume.

**TABLE A5:** Treatment plan clinical objectives for standard and boost plans for anal and rectal cancers

	Clinical objectives					
	Rectum			Anus		
	Target volume/OAR	Objective	Goal	Target volume/OAR	Objective	Goal
Standard plan	PTV	V42.75 Gy (95%)	>99.5%	PTV	D99.5%	≥95% (50.54 Gy)
		V47.25 Gy (105%)	<0.1%		D50%	≥99% (52.6 Gy) and ≤101% (53.7 Gy)
		D50%	≥99% (44.55 Gy) and ≤101% (45.45 Gy)		D1%	≤105% (55.9 Gy)
	Bowel cavity	V20Gy	<400 cc	PTVN − PTV	D99.5%	≥95% (47.9 Gy)
		V30Gy	<250 cc		D50%	≤105% (55.9 Gy)
		V43Gy	<200 cc	PTVE − (PTV+ PTVN)	D99.5%	≥95% (38.0 Gy)
	Bladder	V35Gy	<45%		D50%	≤110% (44.0 Gy)
	X			Small bowel	D200cc	≤35 Gy
					D150cc	≤40 Gy
					D20cc	≤50 Gy
					D5cc	≤55 Gy
				Bladder	D50%	≤45 Gy
					D35%	≤50 Gy
					D5%	≤55 Gy
				Femoral heads	D50%	≤45 Gy
					D35%	≤50 Gy
					D5%	≤55 Gy
				Genitalia	D50%	≤35 Gy
					D35%	≤40 Gy
					D5%	≤55 Gy
Boost plan objectives	GTVBoost	V52.25 Gy (95%)	>99.5%	GTVBoost	D99.5%	≥95% (58.1 Gy)
		V52.25 Gy (105%)	<0.1%		D50%	≥99% (61.0 Gy) and ≤101% (62.2 Gy)

Abbreviations: GTVBoost, boost gross tumor volume; PTV, primary planning target volume; PTVE, elective planning target volume; PTVN, nodal planning target volume.

**TABLE A6:** Organ volumes on computed tomography (CT) and magnetic resonance (MR) for anal and rectal cancer patients including: Mean (range, SD)

		CT (cc)	MR (cc)	Difference (cc)
Rectum	Bladder	275 (48–962, 170)	305 (32–746, 167)	30 (−266–458, 169)
	Sigmoid	108 (23–309, 76)	92 (10–310, 77)	−16 (−126–28, 34)
	Small bowel	264 (0–741, 179)	225 (0–659, 148)	−30 (−406–202, 125)
	Penile bulb	6 (2–11, 2)	6 (2–11, 2)	0 (0, 0)
	Vagina	18 (9–41, 9)	15 (8–31, 7)	−3 (−10–1, 3)
	Uterus	61 (9–116, 31)	57 (22–108, 28)	−4 (−20–15, 10)
Anus	Bladder	318 (95–813, 186)	324 (44–891, 202)	6 (−180–322, 13)
	Sigmoid	156 (36–381, 97)	127 (14–366, 84)	−29 (−99–61, 43)
	Small bowel	284 (27–818, 212)	212 (50–527, 143)	−72 (−292–136, 109)
	Penile bulb	5 (2–11, 3)	5 (2–11, 3)	0 (0, 0)
	Vagina	13 (8–25, 6)	12 (5–18, 5)	−2 (−7–2, 3)
	Uterus	34 (13–64, 16)	31 (14–55, 12.5)	−3 (−9–6, 5)
	Genitalia	298 (58–686, 181)	297 (56.8‐686, 181)	0 (0, 0)
